# CD4^+^ T Cell Priming as Biomarker to Study Immune Response to Preventive Vaccines

**DOI:** 10.3389/fimmu.2013.00421

**Published:** 2013-12-04

**Authors:** Annalisa Ciabattini, Elena Pettini, Donata Medaglini

**Affiliations:** ^1^Laboratorio di Microbiologia Molecolare e Biotecnologia (LA.M.M.B.), Dipartimento di Biotecnologie Mediche, Università di Siena, Siena, Italy

**Keywords:** T cell priming, vaccination, CD4^+^ T cells, mucosal immunity, adoptive transfer, MHC class II tetramers

## Abstract

T cell priming is a critical event in the initiation of the immune response to vaccination since it deeply influences both the magnitude and the quality of the immune response induced. CD4^+^ T cell priming, required for the induction of high-affinity antibodies and immune memory, represents a key target for improving and modulating vaccine immunogenicity. A major challenge in the study of *in vivo* T cell priming is due to the low frequency of antigen-specific T cells. This review discusses the current knowledge on antigen-specific CD4^+^ T cell priming in the context of vaccination, as well as the most advanced tools for the characterization of the *in vivo* T cell priming and the opportunities offered by the application of systems biology.

## Introduction

T cell priming is an essential event for the induction of the adaptive immune response to vaccination. T cell priming is influenced by the type of vaccine formulation (antigen, adjuvant, delivery system), the dose and the route of administration. The characterization of T cell priming induced by a vaccination strategy is therefore critical in order to develop optimal prime-boost combinations capable of eliciting the type of immune response required to fight a specific pathogen.

The efficacy of most preventive vaccines relies on antibody response to block pathogen infection and generation of immune memory cells capable of rapid and effective reactivation following pathogen re-exposure (1, 2). In this context, primary activation of T-helper cells that are required for the induction of high-affinity antibodies and immune memory is essential (2). Furthermore, CD4^+^ T cell priming has been shown to be an early predictor of vaccine immunogenicity in humans (3, 4).

A limitation in the study of *in vivo* T cell priming is due to the low frequency of antigen-specific T cells. This has been overcome by the application of technologies such as adoptive transfer of transgenic antigen-specific T cells into recipient mice and major histocompatibility complexes (MHCs) class II tetramers (5, 6). It is particularly attractive to also consider systems biology approaches that have been recently applied to vaccinology to model T cell priming and develop tools to predict vaccine responsiveness and efficacy (7–9).

Here we review the current knowledge on antigen-specific CD4^+^ T cell priming in the context of prophylactic vaccination. Immunological events following primary vaccination by systemic and mucosal routes and their relevance for the rational development of prime-boost strategies are addressed. Moreover, the methodologies for studying *in vivo* CD4^+^ T cell priming and the potential of applying systems biology for its modeling are discussed.

## Immune Mechanisms of CD4^+^ T Cell Priming

**Figure 1:**
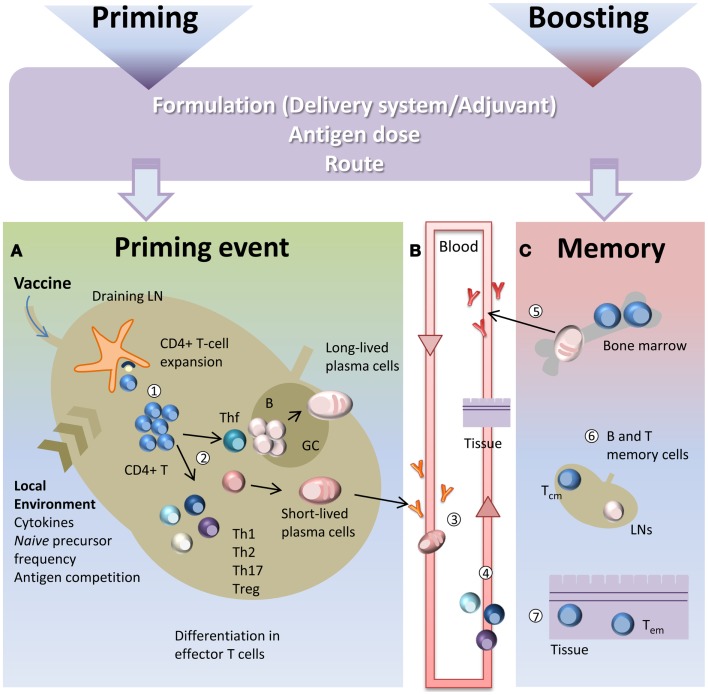
**Immune response triggered by vaccination**. Primary immune response triggered by vaccine administration is influenced by several factors, such as the vaccine formulation (including delivery systems and/or adjuvants), the nature and the dose of the antigen, and the route of immunization. **(A)** After vaccine administration, DCs mature and migrate to the T cell zone of draining LNs. DCs express vaccine epitopes on their MHC class II molecules, thus engaging *naïve* antigen-specific CD4^+^ T cells and inducing their proliferation and differentiation into effector T-helper cells (1). The local environment deeply influences the T cell priming event and the polarization of distinct effector T cell subsets (2). Effector T cells differentiate into subpopulations, such as Th1, Th2, Th17, Treg that mainly exert their function outside the LN. Some primed CD4^+^ T cells differentiate into Tfh that relocate to B-T cell borders. Cognate contact between Tfh cells and antigen-primed B cells in the extra-follicular regions of the lymph nodes is required for clonal expansion and antibody class switching (without affinity maturation) of short-lived plasma cells. GC-Tfh drives the GC reaction, in which B cells undergo clonal expansion, isotype switching, affinity maturation, and differentiate into long-lived plasma cells. **(B)** Low-affinity IgM and IgG antibodies produced by short-lived plasma cells during the extra-follicular reaction, appear at low levels in the serum within a few days after immunization (3). Effector Th1, Th2, and Th17 subpopulations exit the LN and through the blood disseminate toward other LNs and toward the inflamed tissue (in this context, the site of vaccine inoculation) where exert their effector function (4). **(C)** The long-lived plasma cells exit the LN at the end of the GC reaction and migrate to survival niches mostly located in the bone marrow (BM) where they survive through signals provided by supporting stromal cells and continue to release hypermutated antibodies (5). Another fraction of B cells, matured during the GC reaction, develop a memory phenotype and disseminate into the extra-follicular areas of the LN where they persist as resting cells until booster immunization or pathogen encounter (6). Memory T cells traffic through T cell areas of secondary LNs and BM (Tcm) (6), or localize within tissue (Tem) (7). Booster immunization induces a rapid reactivation of memory B and T cells, with proliferation and differentiation into effector cells. Memory B cells mature into plasma cells secreting large amounts of high-affinity antibodies that may be detected in serum within a few days after boosting.

CD4^+^ T cell priming represents a key step in the vaccination process due to the close relationship between CD4^+^ T cells and both long-term humoral immunity and protective antibodies. CD4^+^ T cell priming is influenced by several factors such as the local pro-inflammatory environment, the nature and the dose of the antigen, the vaccine formulation including the type of adjuvant and the route of immunization (10, 11). A schematic representation of the T cell priming event in the context of vaccination is reported in Figure 1. Generation of primed T-helper cells requires contact between antigen-bearing dendritic cells (DCs) and specific CD4^+^ T cells within the T zone of the lymph node (LN) closest to the site of vaccination (2, 12). The process of CD4^+^ T cell priming begins when *naïve* cells, that constantly transit between the circulatory and lymphatic systems, bind their T cell antigen receptors (TCRs) to foreign peptides loaded on MHCs class II molecules presented by antigen presenting cells (APCs), thus leading to T cell proliferation (13). Antigen persistence and duration of peptide presentation by APCs influence the magnitude of the primary T cell response (14, 15). The very early interaction between antigen-specific T cells and peptide-MHC-bearing APCs within the LN has been described with static and dynamic imaging methods and movies (13, 16, 17). Interaction between APCs and antigen-specific *naïve* T cells takes place within the first 8–20 h and is dependent on the presence of the antigen (13). Activated T cells begin to proliferate and finally, in a later and antigen-independent phase, they expand and differentiate into various functionally defined subsets of effector cells that, depending on the nature of the cytokine milieu generated by innate cells, express specific master transcription factors (18, 19). Polarization of the distinct effector T cell subsets is indeed regulated by the strength of antigenic stimulation, as well as by the cytokines present during priming (20). These polarizing cytokines are derived from the APCs, the responding T cells or bystander cells. Effector T cells can be emigrant lymphocytes such as Th1, Th2, or Th17 that exit the LNs and move to inflamed tissues, regulatory cells (Treg), or T follicular helper (Tfh) cells that relocate to B-T cell borders and interfollicular regions (21–23). Tfh cells are specialized to regulate multiple stages of antigen-specific B cell immunity through cognate cell contact and the secretion of cytokines (21). In the extra-follicular reaction, some antigen-primed B cells, after cognate contact of Tfh cells, undergo a process of rapid differentiation in short-lived plasma cells producing low-affinity antibodies such as IgM and IgG that appear in serum at low concentration a few days after immunization (2, 21). Interaction of Tfh cells with B cells drives the formation of germinal center (GC) a dynamic micro-anatomical structure that supports the generation of B cell activation, antibody class switch recombination and affinity maturation (22, 24). Tfh cells that localize in GCs are referred to as GC-Tfh cells. A fraction of B cells matured during the GC reaction acquires the capacity to migrate toward long-term survival niches located within the bone marrow (BM) from where they can release vaccine antibodies for extended periods. Another fraction includes class-specific affinity-matured memory B cells that are able to rapidly expand and differentiate into plasma cells after antigen re-challenge (25).

Upon primary activation most of the antigen-experienced CD4^+^ T cells are short-lived and undergo apoptotic contraction leaving only a small fraction of competent memory precursor cells to migrate into the BM where they differentiate into long-lived memory cells. The frequency of memory T cells reflects therefore the magnitude of the initial T cell expansion and of its subsequent contraction. Two types of memory T cells have been identified based on their phenotype and function (26). Effector memory T cells (Tem) are circulating or tissue-resident cells and exert their immediate effector function after antigen encounter and mediate site-specific protection, while central memory T cells (Tcm) preferentially traffic through T cell areas of secondary LNs and BM and have a high proliferative potential (26). Their role is to recognize antigens transported by activated DCs into LNs and to rapidly undergo massive proliferation generating a delayed, but very large, wave of effector cells (26, 27). During a primary response memory Tfh cells are also generated. These cells are retained within draining lymphoid sites together with antigen-specific memory B cells and persistent complexes of peptide-MHC II (28). During a booster immunization, vaccine antigens restimulate memory T and B cells that rapidly activate a secondary immune response.

## CD4^+^ T Cell Priming in Vaccination

In the context of vaccination strategies, T cell priming can be evaluated as a target for improving the immune response during vaccination as well as a tool for modulating the quality of the immune response. The nature and the dose of the vaccine antigen, the adjuvant or the vaccine delivery used, the route of immunization and the local environment are all factors that deeply affect the primary activation of CD4^+^ T cells (10, 11).

The development of distinct effector CD4^+^ T cell subsets is determined to a great extent by cytokines present during the T cell priming event that act as powerful polarizing factors (10). APCs express toll-like receptors (TLRs) that recognize distinct and highly conserved pathogen-associated molecules, thus activating a signaling cascade that dramatically impacts the quality and the quantity of the T cell response. This has encouraged the use of TLRs ligands as promising adjuvants (10, 29–33), that can influence the effector fate of antigen-specific primed CD4^+^ T cells (10). CD4^+^ T cell priming has been studied for characterizing the mechanism of action of adjuvants such as alum (34), the CpG ODN (35), the lipopolysaccharide (36) or its derivative-like monophosphoryl lipid A (37), cholera toxin (38), or its B subunit (CTB) (39, 40).

Another aspect to consider is the selection of the route of administration of the vaccine that affects the quality and the localization of the T cell response (41, 42). CD4^+^ T cell priming following immunization by different mucosal routes has been characterized in the murine model (35, 38, 43–45) as discussed in the next section. Recently, we have also demonstrated that the route used for priming, but not for booster immunization, influences the skewing of the CD4^+^ T effector response toward Th1 or Th2 with a stronger Th1 polarization upon nasal administration compared to the systemic one (46).

The development of vaccination approaches aimed at enhancing Tfh primary response is particularly attractive. The interaction of T-B cells is stabilized by adhesion molecules, such as the signaling lymphocytic activation molecule (SLAM) family, that initiate intracellular signaling via recruitment of specific adapters such as the SLAM/associated protein (SAP) family (47). Targeted manipulation of the SAP/SLAM family has been employed recently as strategy for shaping and strengthening the immune response during vaccination (47, 48). The employment of a nanoparticle delivery system has also recently been shown to promote robust GC formation and enhance the expansion of vaccine antigen-specific Tfh cells leading to an enhanced humoral response (49).

The role of CD4^+^ T cells in developing durable functional neutralizing antibody responses, via Tfh cells, is considered of key importance for the development of vaccines against pathogens for which no vaccine is currently available, such as HIV (50). Despite the central role of T-helper cells in vaccine immunity, the specific contribution of HIV-specific CD4^+^ T cells in HIV infection is largely unknown and these cells have mostly been excluded from HIV vaccine design strategies because they can be infected by the virus itself (50). Strikingly, in simian immunodeficiency virus (SIV)-infected non-human primates, Tfh cells did not seem to be preferentially infected by the virus, and their frequency in LNs correlated with the magnitude of the SIV-specific IgG response, the avidity of the SIV-specific antibodies and the generation of the GCs (51).

Studies of H5N1 influenza vaccination of healthy adults have shown an increase in the frequency of virus-specific CD4^+^ T cells measured 22 days after the first dose. This increase predicted a rise in neutralizing antibody concentrations after boosting as well as their maintenance 6 months later (3, 52), thus suggesting that primary CD4^+^ T cell response can be considered a predictor marker of the secondary immune response. Similarly, CD4^+^ T cell expansion has shown to predict neutralizing antibody response to monovalent inactivated influenza A H1N1 vaccine (4).

## Mucosal CD4^+^ T Cell Priming

**Figure 2:**
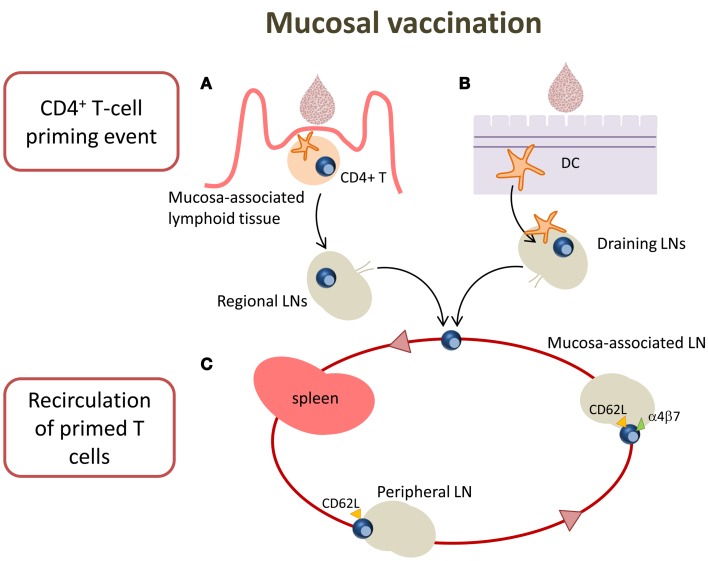
**T cell priming in different mucosal sites following mucosal vaccination**. Mucosal vaccination targets the epithelium that covers mucosal surfaces. **(A)** In many mucosal sites, such as the gastro-intestinal and respiratory tracts, underneath the epithelium barrier inductive sites are present, constituted by organized lymphoid tissue. Following vaccination, antigen is sampled by local DCs and transported into the inductive site where antigen-specific T cell priming occurs. Activated T cells migrate from the inductive site toward the regional draining LNs and then enter into the circulatory torrent through the lymphatic system. **(B)** Vaginal mucosa is devoid of histologically demonstrable organized mucosa-associated lymphoid tissue, therefore after immunization, the antigen is sampled by tissue-resident DCs and transported into the draining iliac LNs that constitute the inductive site. Primed T cells exit the LNs and migrate through the lymphatic system into the blood. **(C)** A fraction of mucosally primed T cells transiently circulates through the blood into the spleen and disseminates into non-draining LNs; the entry into peripheral LNs is CD62L-dependent, while into mesenteric LNs depends on both CD62L and α4β7 expression.

Targeting mucosal sites by vaccination is an important goal considering that over 90% of infections occur at or through mucosal surfaces. The induction of mucosal immune responses requires the presence of a mucosa-associated lymphoid tissue that provides a continuous source of B and T cells to mucosal effector sites (53). A schematic representation of T cell priming in different mucosal sites following mucosal vaccination is reported in Figure 2. Inductive sites for mucosal immunity consist of organized mucosa-associated lymphoid tissue as well as local and regional draining LNs, whereas the effector sites include different compartments mainly consisting of the lamina propria of various mucose (54). Inductive sites in the gastro-intestinal and respiratory tracts have been well defined, and are composed by aggregated lymphoid tissues (gut-, nasal-, and bronchial-associated lymphoid tissues, respectively) and mucosa-associated LNs (mesenteric and mediastinal LNs). On the contrary, the vaginal mucosa is devoid of histologically demonstrable organized mucosa-associated lymphoid tissue and the role of inductive site is played directly by draining iliac LNs (55). Moreover, antigen-uptake across the vaginal mucosa barrier and immune responses in the genital tract are greatly regulated and influenced by the hormonal state and estrus phase (56). Female genital tract has therefore some unique features that should be taken in consideration in the development of a vaccination strategy.

By using the adoptive transfer system (described in the next section), our laboratory has deeply analyzed the CD4^+^ T cell priming following nasal and vaginal immunization in the mouse model. Intranasal immunization with the recombinant vaccine vector *Streptococcus gordonii* (57–62), elicited an early clonal expansion of antigen-specific CD4^+^ T cells in the nasal-associated lymphoid tissue (NALT), and cervical and mediastinal LNs 3 days after immunization (43–45). Proliferated T cells were CD44^hi^CD45RB^lo^ and expressed CD69 molecule within the early cell generations (44). Divided T cells disseminated in the respiratory (44), genital, and intestinal tracts (43) where they become detectable 5 days after priming. Similar results of antigen-specific clonal expansion and dissemination were observed immunizing with soluble ovalbumin (OVA) plus the adjuvant CpG ODN (35, 46). We also demonstrated that homing of nasally primed T cells into distal peripheral LNs was CD62L-dependent, while entry into mesenteric LNs depended on both CD62L and α4β7 expression (35) (Figure 2).

T cell priming was also studied following vaginal immunization in hormone synchronized mice, showing a very efficient activation of CD4^+^ T cells (38, 63). Antigen-specific CD4^+^ T cell clonal expansion was indeed detected in iliac LNs, and proliferated T cells disseminated toward distal LNs and spleen, similarly to what observed following nasal immunization (38). These data show that vaginal immunization is efficient in eliciting CD4^+^ T priming despite the absence of an organized mucosa-associated inductive site in the genital tract (Figure 2).

## Prime-Boost Approach

Characterization of the magnitude and quality of the T cell priming elicited by a vaccine formulation is critically important for the rational development of prime-boost vaccine combinations. An interesting approach to vaccination is indeed the heterologous prime-boost strategy that primes the immune system to a target antigen delivered by a vector and then selectively boosts the secondary response only to the vaccine antigen by using a different vaccine formulation. The heterologous prime-boost approach is specifically aimed at the generation and enrichment of high avidity T cells specific for the target antigen (64). Boosting with a different vector carrying the same antigen has been shown to be more efficient in inducing the immune responses compared to boosting with the same vector. The heterologous prime-boost approach is currently exploited in human studies aimed at developing vaccines against pathogens such as HIV (65), tuberculosis (66), and malaria (67). Furthermore, mucosal and parenteral routes can be combined in a vaccination prime-boost strategy to induce immune responses in both the local and systemic compartments. This approach has shown to be as strong or stronger than those resulting from homologous mucosal or parenteral vaccination alone (68–71). Recently, we have demonstrated that the polarization of CD4^+^ T effector cells is affected by the route used for priming but not for boosting, while local effector responses are mainly dependent on booster route (46).

Recent studies in the mouse model have also assessed the role of peptide-based priming on the subsequent B cell response elicited by whole protein boosting (72) or by infection with the pathogen (73) or with an attenuated viral vaccine (74). These studies showed that CD4^+^ T cell help is quite selective for the subsequent antibody production. CD4^+^ T cells specific for an epitope provide the appropriate help mainly to the protein-specific B cells, indicating a deterministic linkage between antibodies and CD4^+^ T cell responses (73, 74), even if discordant results have been recently reported (75).

Understanding the priming mechanisms of a vaccine formulation and optimizing its priming properties is therefore of critical relevance for the informed design of next generation prime-boost strategies.

## Tools to Study T Cell Priming

Antigen-specific primary activation has been mostly analyzed in animal models, within LNs draining the inoculation site or in the spleen. Primed CD4^+^ T cells can be detected in draining LNs within a few days after immunization, with a peak after 5–7 days (13, 43, 76). In humans, primed T cells can be studied in peripheral blood starting from 7 days following vaccination (4).

Several procedures have been employed to characterize antigen-specific primed T cells, including assays of helper cell activity using carrier/hapten systems (77), and the commonly used proliferation and cytokine production assays. These methods measure functional parameters as a read-out for T cells which react to the specific antigen challenge *in vitro*. A major limitation of these assays is that the phenotypic and functional properties of the reactive cells may be altered by the *in vitro* antigenic restimulation (78).

To overcome this limitation, technologies such as the adoptive transfer of TCR-transgenic T cells in mice (79) and, more recently, MHC class II tetramers (6) have been developed to allow the *ex vivo* analysis of primed T cells (see below). A summary of the most used assays for studying T cell priming in human and animal studies, with their main advantages and disadvantages, is reported in Table 1.

**Table 1:** **Methods used for studying antigen-specific T cell priming in humans and animals**.

Analysis	Assay	Cellular function	Technical methodology	Advantages	Disadvantages
*In vitro*	Proliferation	Cell proliferation	^3^HTdR incorporation	Wide response not restricted to single epitope; high sensitivity	Restimulation and expansion *in vitro*; use of radioisotopes
			Limiting dilution assay	Detection of rare specific T cells	Restimulation and expansion *in vitro;* use of radioisotopes; labor intensive
			Colorimetric assays	No use of radioisotopes	Restimulation and expansion *in vitro*; low sensitivity
	Cytokine release	Cytokine secretion	ELISPOT	Selective identification of distinct cytokine producing cell subsets	Restimulation; identifies only cytokine secreting cells; no phenotypic characterization
			FACS staining	Phenotypic analysis	Restimulation; identifies only cytokine secreting cells; low sensitivity, lethal cell fixation
*Ex vivo*	Adoptive transfer^a^	Cell proliferation	FACS staining	No restimulation; phenotypic analysis of cell generations; localization of labeled cells	Few transgenic mouse strains available; altered physiological condition; laborious procedure
	MHC II tetramers	Enumeration of Ag-specific cells	FACS staining	No restimulation; analysis in physiological condition; phenotypic analysis; rapid analysis; independent from T cell function; high specificity	Peptides have to be predefined; complicated manufacturing; restricted to single epitope specificities

*^a^ Only in mice*.

### Adoptive transfer of transgenic antigen-specific T cells

In order to overcome the limitation of the low frequency of antigen-specific T cells *in vivo*, Jenkins and colleagues developed the adoptive transfer model of antigen-specific transgenic T cells into recipient mice (79). This system largely increases the number of antigen-specific *naïve* CD4^+^ T cells *in vivo* by employing TCR-transgenic mice that express a TCR specific for a defined peptide/MHC complex on most T cells, and thus allows the *ex vivo* analysis of their clonal expansion following antigenic stimulation (5). In order to track their proliferation, transgenic T lymphocytes are labeled with the vital dye 5-(and -6)-carboxyfluorescein diacetate succinimidyl ester (CFSE) (80) and then injected intravenously into immunocompetent recipient mice. Following vaccine administration, the T cell proliferation in the secondary lymphoid organs can be studied by flow cytometric analysis of CFSE dilution in the single-cell generations. The adoptive transfer method has proven to be a powerful tool for studying T cell primary responses to parenteral and mucosal immunization (35, 38–40, 43–45, 81–86), the role of the microenvironment for initiating T cell response in secondary lymphoid tissues (87) and the impact of aging on cellular immunity (88). Transgenic mice extensively used for studying the development of CD4^+^ T cell primary activation following immunization include DO11.10 (89) and OT-II (90) strains that contain rearranged TCR-Vα and -Vβ genes in the germline DNA encoding a TCR specific for chicken OVA peptide_323–339_ bound to I-A molecules in a context of H-2^d^ and H-2^b^ haplotype, respectively. Other transgenic models have been developed, such as SM1 RAG-2 deficient mice, that allow the visualization of *Salmonella* flagellin-specific CD4^+^ T cell responses (91), SMARTA transgenic mice, that produce CD4^+^ T cells expressing Va2 and Vb8.3 TCR specific for the lymphocytic choriomeningitis virus (LCMV) epitope gp61–80 (92), and Ag85B_241–255_ TCR-transgenic mice that allow to characterize tuberculosis-specific immune response (86).

Despite the important results obtained with this system, it has the limitation that the high number of *naïve* antigen-specific T cells transferred into recipient mice alters the physiologic conditions and can influence the immune response observed. Moreover, the study of the antigen-specific primary response is limited by the availability of the transgenic mouse strains with the TCR specific for a given model antigen.

### MHC class II tetramers

The limitations of the methodology described above have been overcome with the development of MHC-peptide complexes. In 1996, the first work describing the use of a peptide-MHC class I complex for the identification and characterization of antigen-specific T lymphocytes was published (93). Initially developed for the study of CD8^+^ T cells, the technology has been extended to the class II system in the context of CD4^+^ T cells (6, 94) and has been applied to the study of human and murine T cells (95–97). This tool has provided an invaluable way to monitor T cell mediated immune responses and quantify the development of an antigen-dependent response. The technology allows identification of antigen-specific T cells based on the specificity of their surface TCR for particular MHC-peptide complexes. Since the affinity of TCR for a single peptide-loaded MHC molecule is generally low, multimerization of the peptide-MHC complexes is necessary for achieving much higher avidities for the TCR (93, 97). Today, the most prevalent multimer used consists of biotin-labeled peptide-MHC complexes bound to streptavidin molecules forming tetravalent structures (96). The peptide of interest can be covalently linked to the β-chain of the MHC molecule for the generation of MHC molecule-peptide complexes or it can be loaded on empty soluble class II molecules (95). Tetramer technology offers the advantage of phenotyping the antigen-specific cells by combining surface marker labeling and allows for the simultaneous detection of different antigen-specific CD4^+^ T cells by using multiple tetramers conjugated to different fluorescent molecules. The major limitations in the use of tetramers are that immunodominant peptides have to be predefined and that humans have very diverse HLA class II molecules. Moreover, the low frequency of antigen-specific CD4^+^ T cells in blood (generally 1/3000–30000) and the low avidity of TCR-MHC-peptide complex recognition are challenging issues for the tetramer technology. One strategy developed to overcome the first problem is the selection of the tetramer-positive cells by sorting with magnetic beads, so the antigen-specific population could be enriched as much as 10,000-fold (76, 98).

By using distinct MHC class II tetramers, Jenkins and colleagues have analyzed in the mouse model the primary response of CD4^+^ T cells specific for three different peptides [the protein FliC_427–441_ of *Salmonella typhimurium*, the OVA_323–339_, and the 2W1S variant of I-Eα protein_52–68_ (2W)] following intravenous immunization and correlated the primary response to the frequency of the respective *naïve* population size by combining the tetramer staining to the magnetic bead enrichment (76). Since the frequency of the *naïve* pool of 2W-specific CD4^+^ T cells in C57BL/6 mice, was the highest among the three peptides assessed, this peptide has been selected for its expression on *Listeria monocytogenes* and *Leishmania major* in order to track, by mean of the 2W-MHC class II tetramer, the peptide-specific T cell primary response following acute infection (99, 100). MHC class II tetramers have been used for identifying CD4^+^ T cell epitopes of *Porphyromonas gingivalis* proteins following oral infection of mice and as a tool for tracking and phenotyping specific effector and memory CD4^+^ T cells (101). In other murine studies, the magnitude and quality of the CD4^+^ T cell response induced by oral immunization with lipid-formulated BCG has been analyzed by using *Mycobacterium tuberculosis* Ag85B_280–294_-specific MHC class II tetramers, and compared with that induced by the subcutaneous immunization with BCG (102).

### Systems biology approach for studying T cell priming

Mathematical and computational modeling can be employed as tool for integrating experimental data into a quantitative analysis of immune responses to antigens. Application of systems biology in vaccinology, named systems vaccinology, has recently been proposed as new powerful tool to model and characterize immune responses to vaccination and to predict vaccine immunogenicity and efficacy (8, 9). Systems vaccinology aims to model the immunological network, from molecules to cells to tissues, in order to predict vaccine immunogenicity. The identification of molecular signatures induced early after vaccination which correlate with and predict the later development of protective immune responses, represents a strategy to prospectively determine vaccine efficacy. Systems biology approaches provide a detailed level of investigation to better and fully analyze the network of interactions within vaccine-specific innate and adaptive immunity. All this information is expected to high impact on rational vaccine development, providing molecular prediction markers of vaccine immunogenicity, uncovering new correlates of vaccine efficacy, as well as guiding the design of new vaccine formulations and prime-boost strategies (7).

Systems biology represents therefore an attractive tool for studying T cell priming and modeling the initiation of the immune response following vaccination and predicting the priming properties of different vaccine formulations. The application of mathematical models can indeed be highly relevant to analyze antigen-specific T cell primary clonal expansion, based on the dilution of the CFSE dye. Quantitative analysis of T cell proliferation through mathematical models has been previously employed for *in vitro* studies of lymphocyte proliferation (103–105). On the contrary, the application to *in vivo* analysis raises several difficulties, mainly due to the fact that a LN is not an “isolated” site but is part of the complex immunological system.

Our group has recently employed a Multi-type Galton–Watson branching process with immigration (63, 106) to model *in vivo* CD4^+^ T cell priming and estimate the probabilities of a cell to enter in division, rest in quiescence or migrate/dye. This model has been successfully applied to analyze CD4^+^ T cell priming in mice immunized by different mucosal routes, such as vaginal or nasal, and has allowed the estimation of the probability of CD4^+^ T cells to enter into division within the draining LNs (63). Ongoing work is focused on modeling lymphocyte trafficking within the lymphatic systems, including both draining and distal LNs and spleen, in order to obtain further quantitative information and generate a model capable of predicting the amount and distribution of primed CD4^+^ T cells.

## Concluding Remarks

CD4^+^ T cell priming is an early biomarker of vaccine immunogenicity that should be considered as a critical parameter in the evaluation of vaccination strategies. The advances in understanding CD4^+^ T cell priming and the availability of latest generation technologies for its study open the way for its use in the rational design of vaccine formulations and prime-boost combinations. CD4^+^ T cell priming can be also considered an important biomarker for early prediction of vaccine immunogenicity and individual responsiveness to vaccination. The application of systems biology and mathematical modeling to the study of CD4^+^ T cell priming offers further opportunities to identify early signatures of vaccine immunogenicity and guide the design of next generation vaccines.

## Conflict of Interest Statement

The authors declare that the research was conducted in the absence of any commercial or financial relationships that could be construed as a potential conflict of interest.
